# Strategies to Reduce the Expert Supervision Required for Deep Learning-Based Segmentation of Histopathological Images

**DOI:** 10.3389/fmed.2019.00222

**Published:** 2019-10-15

**Authors:** Yves-Rémi Van Eycke, Adrien Foucart, Christine Decaestecker

**Affiliations:** ^1^Digital Image Analysis in Pathology (DIAPath), Center for Microscopy and Molecular Imaging (CMMI), Université Libre de Bruxelles, Charleroi, Belgium; ^2^Laboratory of Image Synthesis and Analysis (LISA), Ecole Polytechnique de Bruxelles, Université Libre de Bruxelles, Brussels, Belgium

**Keywords:** histopathology, deep learning, image segmentation, image annotation, data augmentation, generative adversarial networks, transfer learning, weak supervision

## Abstract

The emergence of computational pathology comes with a demand to extract more and more information from each tissue sample. Such information extraction often requires the segmentation of numerous histological objects (e.g., cell nuclei, glands, etc.) in histological slide images, a task for which deep learning algorithms have demonstrated their effectiveness. However, these algorithms require many training examples to be efficient and robust. For this purpose, pathologists must manually segment hundreds or even thousands of objects in histological images, i.e., a long, tedious and potentially biased task. The present paper aims to review strategies that could help provide the very large number of annotated images needed to automate the segmentation of histological images using deep learning. This review identifies and describes four different approaches: the use of immunohistochemical markers as labels, realistic data augmentation, Generative Adversarial Networks (GAN), and transfer learning. In addition, we describe alternative learning strategies that can use imperfect annotations. Adding real data with high-quality annotations to the training set is a safe way to improve the performance of a well configured deep neural network. However, the present review provides new perspectives through the use of artificially generated data and/or imperfect annotations, in addition to transfer learning opportunities.

## 1. Introduction

**Figure 1:**
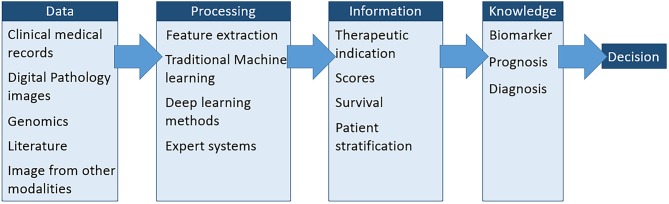
The different steps implemented in computational pathology. These steps aim to extract the most accurate information possible from all available data to improve complex diagnosis and therapeutic decisions (2).

More and more information is needed for diagnosis and therapeutic decision-making, especially in the context of “personalized medicine.” As a result, pathologists are expressing a growing demand for the automation of their most recurrent tasks and for a more complex set of analyses required for their research activities. It has therefore become crucial to integrate unbiased quantitative assessments into pathologist's practice and research. For this purpose, whole slide imaging enables automated image analysis with multiple advantages, such as the objective evaluation of morphological and molecular tissue-based biomarkers. Much progress has been made on slide scanner devices. Hence, less than 30 s are necessary to scan a 10 × 10 mm^2^ at 40x with the newest devices^1^. The resolution and quality that can be obtained through this process are now comparable to the resolution of a standard light microscope. An additional interesting feature is the ability to produce a sharp image from scans performed at different z-levels, a process also known as z-stacking. This feature prevents blurring on thick samples or enables to identify very thin signals such as the small dots produced by *in situ* hybridization. Whole slide imaging is now involved in a growing number of developments and applications in various fields covering basic science, pathology, and pharmaceutical research. With the development of “personalized medicine,” the data relating to each patient or population are exploding. Fortunately, the computer storage and computing power is increasing. In this context, the concept of “digital pathology” is shifting to that of “computational pathology.” This latter approach “integrates multiple sources of raw data (e.g., clinical electronic medical records, laboratory data, including ‘omics,' and imaging)” (1). Figure 1 summarizes the different steps of this approach. In addition to biomarker evaluation, computational pathology aims to characterize a disease at the molecular, individual and population levels. This approach also transforms those data into knowledge that can be directly used by pathologists and clinicians.

An important contribution to computational pathology is computational histology or “histomics,” which aims to extract as much information as possible from digital histological slides (3). Histomics makes it possible to characterize the histological manifestation of a disease by taking into account the morphological, spatial and microenvironmental context. Image analysis plays a key role in histomics. In this context, deep learning provides new ways to extract information more efficiently from raw data, in general, and from images, in particular. A significant contribution to histomics is brought by the development of challenges during biomedical imaging conferences. During those challenges, image experts are confronted with complex image analysis problems. Since 2013, the number of such challenges rocketed. In recent ones, deep learning totally outperformed the classical image analysis approach. For example, the Camelyon17^2^. Deep neural networks have also been applied to tumor grading (4), cancer diagnosis (5), and prognosis (6). Interestingly, recent studies also suggest that genetic traits can be inferred from histological features (3, 7). However, deep learning is known to be a data-hungry method, requiring much more training data than standard machine learning approaches (8). Collecting such data for histomics applications can be problematic, particularly for image segmentation, which requires manual annotations from pathologists, a rare and expensive resource. Histological structure segmentation is involved in different key applications in histopathology, such as the extraction of morphological measurements for tumor grading or the possibility to evaluate immunohistochemical biomarkers in specific compartments (e.g., tumor vs. stroma). For this purpose, pathologists have to annotate thousands of structures present in histological slide series, a long, tedious, and potentially biased task that would greatly benefit from automation.

The present paper aims to review strategies that could help provide the very large number of annotated images needed to automate the segmentation of histological images using deep learning. The following sections describe four different approaches that we identified: the use of immunohistochemical markers to label cells of interest, realistic data augmentation, Generative Adversarial Networks (GAN)—another deep learning method that is able to generate artificial examples—and transfer learning. In addition, we describe alternative learning strategies that are able to cope with imperfect annotations, another way to reduce the experts' workload in image annotation. We then discuss tasks that remain to be done to make the most of these strategies to minimize the need for expert supervision.

## 2. Use of Immunohistochemical Markers to Label Cells of Interest for H&E Image Segmentation

Immunohistochemistry (IHC) and special colorations, such as Goldner, are incredible technologies which allow highlighting certain cell types or structures of interest. For instance, pan-cytokeratin (AE1/AE3) is specifically expressed by the epithelial cells in most epithelia and their tumors, with some exceptions (e.g., in case of epithelial-to-mesenchymal transition) (9). This kind of marker can be thus used to create a precise and objective ground truth. A recent and interesting study on prostate tissue analysis illustrates this approach (10). Tissue sections were stained with H&E and digitized. They were subsequently destained and restained with P63 and CK8/18 IHC markers to highlight epithelial structures and also digitized. After registering the IHC and H&E image pairs, segmenting the stained structures on an IHC image enabled to produce a binary mask, i.e., an image whose pixels only have two values: 1 for pixels at the locations of objects of interest and 0 for other pixels. This mask can then be applied as an annotation on the corresponding and registered H&E image. The resulting annotated H&E images can then be used to train a deep network to segment the structures of interest on H&E prostate tissue sections. In this study the authors also tried to improve the performance by correcting mask alterations due to some staining artifacts, such as those resulting from structures and debris inside the glands. To avoid these alterations the authors propose to train a deep network to produce correct masks from IHC images. This approach required to provide staining masks corrected manually to the system. These corrections took much less time than providing the complete annotation manually but the obtained result improvement is relatively low. Previously, the same team used PHH3 restaining as a standard reference for automatizing mitosis identification in H&E images from breast cancer sections (11). Of course, this kind of approach is limited to targets that can be specifically identified by antibodies or special staining. It should be noted that such an approach is facilitated when there is close collaboration between the people in charge of tissue processing and those in charge of image analysis.

## 3. Realistic Data Augmentation

Data augmentation is a technique used in machine learning and deep learning to create artificial data for training. These data can be generated from rules set by the programmer or from actual data that have been altered. Creating artificial data for learning is useful to avoid overfitting and to increase the model's ability to generalize. This approach also aims to expose the model to as many variations as possible that may occur in a specific sample space and thus to enhance its robustness. Data augmentation is therefore also a way to reduce the potential influence of irrelevant sources of variation present in training data.

**Figure 2:**
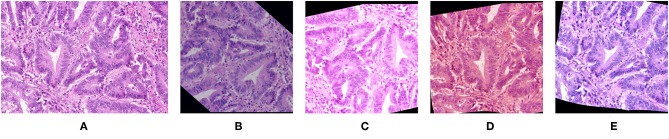
Images generated by a data augmentation strategy. **(A)** The original image and **(B–E)** various images which are generated from **(A)** using a data augmentation strategy described in Van Eycke et al. (20). This strategy combines image alterations targeting color, intensity, geometry, and image quality features, such as sharpness.

In the context of histological image processing, the sample space consists of all images of all tissue samples that are likely to be analyzed with all possible variations regarding staining, acquisition parameters, and possible artifacts. Data augmentation therefore aims to reproduce those variations. Current practices often focus on geometric variations applied to the training images, such as affine transformation (e.g., flip, rotation, and translation) and blurring, in order to make the model invariant for these transforms (12). Recently, Xu et al. showed that the additional use of elastic transformations—modifying tissue morphology—is beneficial for gland instance segmentation (13). Color augmentation is also investigated to take into account stain variations. It usually consists in random transformations applied on standard color representations such as those provided by the RGB or HSV color space [see e.g., (12, 14, 15)], or after extraction of principal (RGB) components (16, 17). It should be noted that random variations in the RGB space should be small to prevent from producing aberrant colors out of the range of the standardly used histological staining techniques (such as H&E and IHC). Concerning principal components, studies on color normalization show that the principal components do not provide an appropriate representation of the color space for H&E (18) and IHC staining (19). Furthermore, the color alterations proposed in previous studies are generally based on linear transformations applied to the whole image without specifically targeting the stained tissue. In a recent work, we propose a more sophisticated approach for realistic “color augmentation” (20). Our approach is based on color deconvolution, a standard method to separate the different staining components (e.g., H&E, or hematoxylin and DAB in IHC). It essentially consists in identifying in the RGB color space the color vector specific to each staining component. Each of them can then be independently altered in terms of orientation to modify the staining color. Transformations at the intensity level complete the possible staining component alterations. Figure 2 illustrates images generated by a data augmentation strategy targeting geometry, color, intensity, and other acquisition-related features (20). The alterations are applied to the training images (and their segmentation masks in the cases of spatial transformations) to increase their number drastically. Different studies clearly evidence the positive impact the different data augmentation components have on deep learning performances (12, 13, 20). Furthermore, realistic data augmentation strategies are able to limit the impact of changes in tissue processing, staining, and image acquisition features, including changes in resolution (20). Table 1 provides a summary of the different methods presented in this section.

**Table 1:** Summary of data augmentation methods.

**References**	**Geometry transforms**	**Color transforms**	**Other**
Xu et al. (13)	Affine + elastic transforms	–	–
Lafarge et al. (14)	Affine transform	Linear transforms on each RGB color channel	–
Sirinukunwattana et al. (15)	Affine transform	Linear transforms in the HSV color space	–
Mishra et al. (16) Xu et al. (17)	Affine transform	Linear transforms on the principal (RGB) components	–
Van Eycke et al. (20)	Affine + elastic transforms	Linear or non-linear (if required) transforms on color channels in the deconvoluted space + linear transforms of the exposure and color temperature	Blurring

## 4. Generative Adversarial Networks (GAN) to Augment Training Data

**Figure 3:**
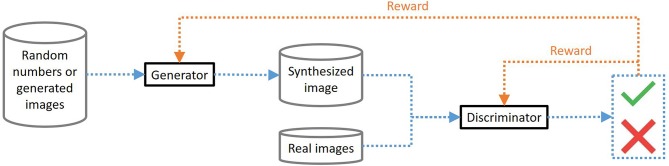
Generative adversarial networks (GAN) principles. Cylinders represent data while black rectangles represent neural networks. The main path appears in blue while the feedback loops appear in orange. The generator receives input data that allows it to synthesize an image. The discriminator receives either a real image or a synthesized image as an input. It must then determine whether it is a real or generated example. The generator is rewarded if it succeeds in deceiving the discriminator while the discriminator is rewarded if it succeeds in distinguishing the true images from the generated images.

Generative adversarial networks (GAN) are algorithms that combine two artificial neural networks, a generator (G) and a discriminator (D) network, to generate realistic artificial data (21). The purpose of the G network is to create more-real-than-life data capable of “deceiving” a human (and an algorithm). Network D is used to judge the reality of the data (instead of a human). The two networks are trained in parallel in a competitive scheme until they converge and reach a Nash equilibrium (22): G is “rewarded” if it manages to fool D, whereas D is rewarded if he can distinguish false images (generated by G) from true ones (see Figure 3). For generating artificial images, the input of G is usually a series of random numbers or images (as explained below), while that of D mixes real images and those provided by G. Once the GAN is trained, new sequences of random inputs are used to generate new realistic images from G.

This type of architecture can be used in several ways and notably to generate examples, a process sometimes called “GAN augmentation.” This process can be considered as an alternative or a complement to standard data augmentation described above. Other applications include the increase in image resolution, image normalization or style transfer, which consist in composing an image with a style (i.e., characteristics such that pattern, color palette, etc.) learned from another set of images. In the present review, we focus on GAN-based data augmentation useful for histological image segmentation. An advantage of this approach on standard data augmentation is that a GAN is able to learn the different sources of variation present in an image set. This learning, or modeling, is then used to generate artificial but realistic images in order to increase a training set (23).

In histopathological imaging, GAN-based approaches can be very useful to take into account complex variations, such as those induced by tumor heterogeneity. These variations are difficult to produce by standard data augmentation techniques. Given a sufficient number of training examples illustrating this heterogeneity, a trained GAN is able to produce new examples that are “intermediate” between training examples. However, for image segmentation supervision must also be generated.

**Figure 4:**
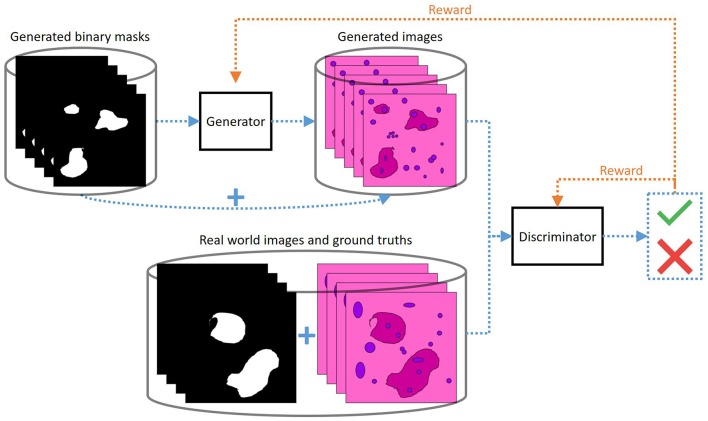
Use of a GAN to generate examples for histological image segmentation (same graphic conventions as in Figure 3). Computer-generated binary images are provided as inputs to the generator to generate images (of the same size) that mimic the targeted tissue structure. The discriminator receives as input a binary image and a tissue image, artificial or real, of which it must determine the origin. The binary images associated with the real images are the segmentation masks of the structures of interest. Therefore, in the generated images the structures of interest must appear at the locations indicated by the white masks in the generator inputs in order to be able to deceive the discriminator. In this way, after system optimization, the binary images provided to the generators correspond to the segmentation masks of the generated images.

The most common way consists in generating binary segmentation masks that mimic true ones observed in the available supervised data and feeding them to the generator as inputs (Figure 4). These binary masks are usually generated using specific algorithms adapted to the targeted tissue/cell structure. For example, in the case of cell nuclei, white discs can be drawn automatically and randomly in a black image (24). The outputs of the generator are expected to be realistic images showing the structures of interest at locations indicated by the input masks. The synthesized images are then fed as inputs to the discriminator together with real images. The masks can also be fed as inputs to the discriminator together with the (generated or real) images to enforce a better consistency between the masks and images (24).

The study by Bowles et al. (23) provides a very good illustration of such methodology applied to medical image segmentation. The authors noted that GAN augmentation provides an efficient tool for interpolating within the training data distribution. However, it cannot extrapolate beyond its extremes without the aid of standard geometric augmentation. These results suggest a synergic effect between standard data augmentation and GAN augmentation. This effect was obtained on very small image sets and remains to be confirmed on histological images. Concerning histological image segmentation specifically, most studies on GAN has focused on cell nucleus segmentation (24–28). Each of them provides slight variations such as the ability to generate more specific images (27), to improve even more the consistency between the mask and the synthesized image (24), or to generate images with positive and/or negative nuclei for IHC staining (28). Concerning the architectures used, the discriminator usually consists of modified versions of classification networks such as the Resnet (29) or the Markovian discriminator (30). The generator usually consists of a modified version of a U-net (31). GAN training usually follows a standard procedure consisting of alternating discriminator and generator training at each step (21). Table 2 provides a summary of the characteristics of the different methods mentioned in this section.

**Table 2:** One-sentence summary of GAN augmentation methods [focusing on cell nucleus segmentation, except the first one (23)].

**References**	**Method**
Bowles et al. (23)	Progressive Growing GAN (with added noise on some layers) using multichannel image patches containing a tissue image and its segmentation mask
Mahmood et al. (24)	Cycle GAN to generate examples with consistent segmentation from unpaired images and masks
Zhang et al. (25)	Conditional GAN with tweaks to generate image patches and their segmentation masks from an original tissue image (not really data augmentation)
Hou et al. (26)	Generation of nuclei-free background and foreground textures separately, using standard image processing techniques, which are then combined into an image that is finally refined using a GAN
Hu et al. (27)	InfoGAN to generate different cell subtypes from unclassified segmented cells
Senaras et al. (28)	Conditional GAN to generate 3 classes of examples for IHC staining (positive cells, negative cells and background)

## 5. Transfer Learning

Transfer learning relates to the use of a model trained on a task as a starting point, i.e., as a pre-trained model, to tackle another different task that does not necessarily relate to the first one [for a general survey, cf. (32)]. This pre-trained model can then be refined using a limited dataset available for the new task of interest. Transfer learning has become very popular with deep learning because of the large amounts of resources required to train models from scratch (33).

In image processing a common application of transfer learning consists first in training a convolutional neural network (CNN) using large public databases of natural images. Then, the last layers of the network are refined using other images specific to the task of interest, e.g., cell nucleus classification (34). This strategy is based on the fact that in a deep neural network, the first layers act as generic feature extractor, whereas the last layers tend to be more task-specific (33). The fine-tuning step using new images requires prior adaptation of the structure of the last CNN layers in order to produce the desired outputs for the task of interest. A variant consists of end-to-end fine-tuning of the whole structure, and not only the last layers, of the pre-trained network (35). The underlying assumption of transfer learning, which is now confirmed by many studies, is that the features learned by CNNs trained on natural images could also be useful for medical ones. Moreover, such pre-trained networks on natural images are publicly available and thus allows to reduce significantly both the time and the number of examples specific to the final task that are required to fine-tune the deep network. Studies show that compared to models trained from scratch, transfer learning improves the robustness and performance of CNNs for medical image processing tasks, including segmentation (35–37). Transfer learning can also be used to fine-tune a model to a specific task close to the task for which it was originally trained. For example, a model that has been trained to segment colorectal glandular epithelium may be re-trained with a minimum number of examples to be able to segment prostate epithelium. Similarly, it is possible to use transfer learning to adapt a network to different acquisition parameters, different structures of interest, different stainings, … with a minimum number of examples (20). Table 3 provides a summary of the different methods presented in this section.

**Table 3:** Summary of transfer learning methods.

**References**	**Pre-training**	**Fine-tuning**
Bayramoglu and Heikkilä (34)	On natural images	The last layers with task-related training examples
Du et al. (35)	On natural images	End-to-end with task-related training examples
Van Eycke et al. (20)	On a task close to the final task	With task-related training examples

## 6. Other Learning Strategies Able to Use Imperfect Annotations

Being less demanding in the quality of annotations is another way to facilitate the collection of large annotated datasets. Being able to use imperfect or imprecise supervision while producing good results is an important challenge for deep learning. For example, in histopathology a pathologist might label whether or not a given histological image includes cancer cells rather than precisely delineating the cancer region. After training on a collection of such inaccurately annotated images, a weakly-supervised learning algorithm could automatically detect and even segment cancerous tissue areas in new images (38).

**Figure 5:**
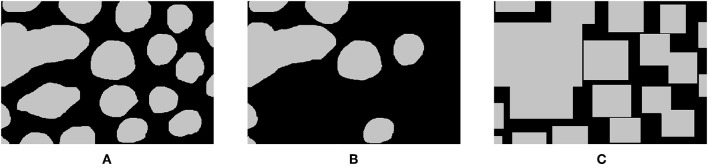
Examples of imperfect annotations generated from high quality ones. **(A)** Original annotations from the GLaS challenge (12), **(B)** noisy annotation where some labels are removed, **(C)** weak annotations based on bounding boxes.

Imperfect annotations can be characterized using well-known paradigms from classical machine learning. In a recent study, we classify these imperfections in three broad categories (39). First, semi-supervised learning describes cases where a large part of the dataset lacks labels, i.e., the number of supervised samples is lower than all available samples. Second, weak learning generally describes a lack of precision in the segmentation label. Instead of the expected pixel-precise segmentation, the labels may apply to approximate shapes, bounding boxes, or an entire image (as mentioned in the example above). Third, the last category covers cases where the accuracy of the annotated pixel class is doubtful, which are denoted as noisy datasets. There is some overlap between those categories. For instance, semi-supervised datasets can also be characterized as noisy, because missing labels can also be considered as objects mistakenly labeled as belonging to the background class. Figure 5 shows illustrations of such imperfect annotations. Using the classical machine learning paradigms allows us to get inspiration from strategies which have already been used to cope with these imperfections, outside of Deep Learning.

Semi-supervised methods typically use a two-step process. First, they estimate the shape of the data distribution from the entire dataset, including unlabeled samples. Then, the labeled data is used to separate the classes within that distribution (40). Weak Learning methods usually follow the framework of Multiple Instance Learning (MIL), where unlabeled instances (i.e., pixels in image segmentation) are grouped into labeled bags (i.e., images) and the task is to predict instance-level label (41, 42). Strategies for managing noisy dataset will often rely on estimating the noise transition matrix, which describes the probability for a given label to be mistaken to another (43). These strategies can all be adapted to Deep Learning methods (44, 45). In particular, the MIL strategy was successfully applied for histological image segmentation by operating typical imperfections in image annotations (38). This work was very recently generalized and extended to other medical image modalities (46). In a systematic study on imperfectly labeled datasets (39), we firstly show that deep learning methods are naturally robust to a certain amount of noise and imprecision in the annotation outlines. Secondly, the performance against highly imperfect supervision is greatly improved by combining semi-supervised, noisy and weak learning strategies. In particular, it is often better to use a smaller data set with fewer annotation problems than a larger one with strong defects. In this latter case, it seems more appropriate to consider those dataset parts with strongly uncertain supervision as unlabeled in a semi-supervised learning paradigm (39).

## 7. Discussion and Conclusion

The literature lacks systematic and comparative studies to draw clear conclusions about the adequacy, contribution and potential synergies of the different techniques described above. Moreover, not all segmentation problems are equivalent, particularly in terms of the size, number and degree of heterogeneity of the objects of interest (e.g., cell nuclei, glands, tumor vs. stroma areas, etc.). It can be expected that the characteristics of the targeted objects have an impact on the strategies to be implemented to overcome supervision deficiencies. Nevertheless, some aspects can be highlighted from the above literature review by crossing some results.

As detailed in the previous section, CNNs seem to be insensitive to small imperfections in annotations (39). Therefore, one can be reassured about the use of IHC markers to identify cells and structures of interest on H&E images via image realignment that can be a source of (small) errors, as can small staining artifacts. The same applies to small variations in the manual annotations of experts.

Concerning transfer learning, using pre-trained CNN on large sets of natural images may be questioned for histological image segmentation tasks. The advantage is that such pre-trained networks are publicly available. However, these networks usually present heavy structures and are initially designed for image classification. An alternative that seems more suitable is to use a network configured for (biomedical) image segmentation, such as U-Net, which is now available via an ImageJ plugin (31, 47). This network can be pretrained on public histological image databases to extract more specific histology-related features, and then fine-tuned on a small set of images related to the targeted task (48). These public databases are notably available via the challenges organized during conferences such as ISBI and MICCAI^3^^,^^4^^,^^5^^,^^6^. Tables 4, 5 provide an overview of the corresponding datasets and the data augmentation strategies used by the best-performing teams in these different challenges. In a recent study, we successfully applied this approach with a new CNN that we pre-trained (from scratch) on the H&E images provided by the MICCAI Gland Segmentation (GlaS) Challenge 2015 (12) and then fine-tuned on a very small set of IHC images from our laboratory (20). However, our results also show that intensive and realistic data augmentation can be able to challenge this kind of transfer learning even with a small amount of training data. Our results also suggest that the same applies for classical transfer learning from natural images (20). Indeed, our data augmentation strategy allowed us to achieve, with full network training from scratch, superior performance on the Glas Challenge dataset than the challenge winner, i.e., the DCAN network, that benefited from pre-training on a wide range of natural images (49).

**Table 4:** Characteristics of the discussed public databases. WSI, whole slide images; ROI, region of interest.

**Database**	**Tissue**	**Staining**	**Training samples**	**Test samples**	**Annotations**
GlaS	Colorectal	H&E	85 images from 16 histological sections	60 images from 16 histological sections	Gland segmentation
Tupac 2016 main dataset	Breast cancer	H&E	500 WSI from the cancer genome atlas	321 WSI	Proliferation scores
Tupac 2016 auxiliary dataset 1	Breast cancer	H&E	656 images from 73 different cases	34 images from 34 different cases	Mitosis locations
Tupac 2016 auxiliary dataset 2	Breast cancer	H&E	148 WSI from the main dataset	321 WSI	Mitosis counts in some ROIs
Camelyon 2016	Mammary (sentinel) lymph node metastasis	H&E	270 WSI from 2 medical centers	130 WSI from 2 medical centers	Metastasis segmentation
Camelyon 2017	Mammary lymph node metastasis	H&E	500 WSI from 100 patients from 5 different centers	500 WSI from 100 patients from 5 different centers	Metastasis segmentation

**Table 5:** For each challenge, best participating teams and a summary of their data augmentation methods.

**Best participating teams**	**Data augmentation methods**
**GlaS challenge**^**a**^ **(colon gland segmentation)**	
CuMedVision	Transfer learning from natural images; affine and elastic (geometry) transforms
ExB	Affine and elastic (geometry) transforms; Gaussian blurring
Freiburg	Affine and elastic (geometry) transforms; random multiplications in HSV color space
**Tupac 2016**^**b**^ **(breast tumor proliferation assessment)**	
Lunit inc.	Image translation; color, brightness, and contrast modifications
Contextvision	Affine (geometry) transforms
Sectra	No information
Radboud UMC	Affine and elastic (geometry) transforms; linear intensity transforms of the deconvoluted color channels; brightness, contrast, and saturation modifications; blurring and additive Gaussian noise
IBM Research	No information
**Camlyon 2016**^**c**^ **(detection of lymph node metastases)**	
HMS and MIT	Image rotation; additive color noise
ExB	Image rotation and mirroring
Q.Wong	Image mirroring
**Camelyon 2017**^**d**^ **(detection of lymph node metastases)**	
Shlee	Affine (geometry) transforms; contrast and HSV color space modifications
Ozymandias.watchman	Image flip and rotations; HSV color space modifications
Ericzz	Affine (geometry) transforms; linear transforms of the RGB color channels and HSV modifications

a https://warwick.ac.uk/fac/sci/dcs/research/tia/glascontest

b http://tupac.tue-image.nl

c https://camelyon16.grand-challenge.org

d https://camelyon17.grand-challenge.org/

In view of the complexity of some of the methods with respect to their effectiveness, here are some recommendations:

1. The use of IHC biomarkers to create segmentation masks can be considered the most effective and accurate approach. It is therefore to be preferred when it is usable.
2. As far as possible, pretrain the network with data close to the final data and for which supervision exists.
3. Data augmentation has proven to be a very effective way to improve performance. For histopathological images, it is preferable to apply at least a mix of geometric and color transforms. The kind(s) of geometric transform (e.g., affine and/or elastic) to be applied depend(s) on the morphological characteristics of the object to be segmented.
4. GANs allow to generate variations that are too difficult to implement with standard augmentation techniques. However, GANs can only interpolate between existing examples. It should be noted that GANs may be subject to instability during training and can have unpredictable behaviors. Given the current state of the art, it is difficult to recommend them as part of a systematic approach.
5. When supervision is very imperfect, the alternative learning methods described in section 6 can be used to extract the best possible information from the segmentation examples. However, having at least a small set of correctly annotated images is strongly recommended and allows data augmentation techniques to be applied. It is preferable to complete with unsupervised data in a semisupervised learning scheme, rather than include highly incorrect or too partial supervision.

In conclusion, adding to the training set real data with high-quality annotations, obtained either from an expert or with the IHC approach described in section 2, is a reasonably safe way to improve the performance of a well configured deep neural network. Even with (slightly) noisy supervision, a logarithmic relationship between performance and the amount of training data can be expected (50). In this context, the present literature review brings new perspectives with the use of artificially generated data and/or imperfect annotations, in addition to transfer learning opportunities. It remains to clarify possible synergies in combining several strategies, such as data and GAN augmentation. In future work, we will also assess the usefulness of such intensive augmentation strategies in cases of relatively imperfect annotations.

## Author Contributions

CD: manuscript design. Y-RV, AF, and CD: drafting the manuscript.

### Conflict of Interest

The authors declare that the research was conducted in the absence of any commercial or financial relationships that could be construed as a potential conflict of interest.

## Footnotes

^1^https://www.hamamatsu.com/jp/en/C13220-01.html

^2^https://camelyon17.grand-challenge.org challenge showed that deep learning was able to effectively analyze information from several tissue slides from the same patient to predict pN stages of breast cancers.

^3^https://warwick.ac.uk/fac/sci/dcs/research/tia/glascontest

^4^http://tupac.tue-image.nl

^5^https://camelyon16.grand-challenge.org

^6^https://camelyon17.grand-challenge.org/
